# Beyond Total Mesorectal Excision (TME)—Results of MRI-Guided Multivisceral Resections in T4 Rectal Carcinoma and Local Recurrence

**DOI:** 10.3390/cancers15225328

**Published:** 2023-11-08

**Authors:** Sigmar Stelzner, Thomas Kittner, Michael Schneider, Fred Schuster, Markus Grebe, Erik Puffer, Anja Sims, Soeren Torge Mees

**Affiliations:** 1 Department of General and Visceral Surgery, Dresden-Friedrichstadt General Hospital, Teaching Hospital of the Technical University of Dresden, D-01067 Dresden, Germany; anja.sims@klinikum-dresden.de (A.S.); soeren-torge.mees@klinikum-dresden.de (S.T.M.); 2 Department of Visceral, Transplant, Thoracic, and Vascular Surgery, University Hospital of Leipzig, D-04103 Leipzig, Germany; 3 Department of Radiology, Dresden-Friedrichstadt General Hospital, Teaching Hospital of the Technical University of Dresden, D-01067 Dresden, Germany; thomas.kittner@klinikum-dresden.de; 4 Department of Urology, Dresden-Friedrichstadt General Hospital, Teaching Hospital of the Technical University of Dresden, D-01067 Dresden, Germany; michael.schneider@klinikum-dresden.de (M.S.); fred.schuster@klinikum-dresden.de (F.S.); 5 Department of Gynaecology, Dresden-Friedrichstadt General Hospital, Teaching Hospital of the Technical University of Dresden, D-01067 Dresden, Germany; markus.grebe@klinikum-dresden.de; 6 Institut of Pathology, Dresden-Friedrichstadt General Hospital, Teaching Hospital of the Technical University of Dresden, D-01067 Dresden, Germany; erik.puffer@klinikum-dresden.de

**Keywords:** pelvic compartments, locally advanced rectal cancer, locally recurrent rectal cancer, MRI assessment, MRI-guided surgery, prognosis

## Abstract

**Simple Summary:**

Surgery for rectal cancer involving adjacent organs (T4 primary tumors) or for locally recurrent rectal cancer requires dissection planes beyond the well-defined perimesorectal space. It is, therefore, of paramount importance to define the extent of surgery preoperatively. Magnetic resonance imaging (MRI) provides adequate guidance for the surgeon to achieve a clear resection margin. In this study, the diagnostic performance of MRI against histopathology and oncological outcomes that can be achieved with MRI-guided surgery are studied using an MRI-based division of the pelvis into seven compartments. Overall, the accuracy of MRI is good, yielding excellent results for T4 tumors and good results for locally recurrent tumors. Complete histopathologic (R0) resection is the most important determinant of outcome.

**Abstract:**

Rectal cancer invading adjacent organs (T4) and locally recurrent rectal cancer (LRRC) pose a special challenge for surgical resection. We investigate the diagnostic performance of MRI and the results that can be achieved with MRI-guided surgery. All consecutive patients who underwent MRI-based multivisceral resection for T4 rectal adenocarcinoma or LRRC between 2005 and 2019 were included. Pelvic MRI findings were reviewed according to a seven-compartment staging system and correlated with histopathology. Outcomes were investigated by comparing T4 tumors and LRRC with respect to cause-specific survival in uni- and multivariate analysis. We identified 48 patients with T4 tumors and 28 patients with LRRC. Overall, 529 compartments were assessed with an accuracy of 81.7%, a sensitivity of 88.6%, and a specificity of 79.2%. Understaging was as low as 3.0%, whereas overstaging was 15.3%. The median number of resected compartments was 3 (interquartile range 3–4) for T4 tumors and 4 (interquartile range 3–5) for LRRC (*p* = 0.017). In 93.8% of patients with T4 tumors, a histopathologically complete (R0(local)-) resection could be achieved compared to 57.1% in LRRC (*p* < 0.001). Five-year overall survival for patients with T4 tumors was 53.3% vs. 32.1% for LRRC (*p* = 0.085). R0-resection and M0-category emerged as independent prognostic factors, whereas the number of resected compartments was not associated with prognosis in multivariate analysis. MRI predicts compartment involvement with high accuracy and especially avoids understaging. Surgery based on MRI yields excellent loco-regional results for T4 tumors and good results for LRRC. The number of resected compartments is not independently associated with prognosis, but R0-resection remains the crucial surgical factor.

## 1. Introduction

The standard of surgical care for patients with rectal cancer is total mesorectal excision (TME)-based surgery with five-year local recurrence rates of approximately 5% and even lower in contemporary surgical series [1,2]. Nevertheless, locally recurrent rectal cancer (LRRC) remains an issue in rectal cancer treatment, and if detected, salvage surgery should be considered as a potentially curative option [3]. In addition, about 10% of patients with rectal cancer present with infiltration of adjacent organs (T4-tumors) [4]. Both LRRC and T4-primary tumors require complex surgical management with en bloc removal of involved organs and structures beyond the well-defined planes of TME surgery [5]. These often exenterative operations may result in considerable morbidity and functional sequelae [6,7]. It is, therefore, of paramount importance to carefully select patients for surgery. MRI has emerged as the gold standard for assessment of the tumor spread in the small pelvis [8,9,10]. However, beyond-TME surgery is rather a surgical strategy than a clearly defined procedure. Therefore, it is common clinical practice to divide the pelvis into compartments and remove those parts that are involved. Surgery is guided by the findings of MRI and depends on its accuracy. This approach enables the surgeon, on the one hand, to achieve a clear surgical margin and, on the other hand, to spare uninvolved compartments and, hence, function. Georgiou et al. established an MRI staging system based on seven pelvic compartments and reported excellent results with respect to diagnostic performance [11]. Meanwhile, the usefulness of the proposed pelvic compartmentation was confirmed on an anatomical base [12].

The aim of this study is to investigate the performance of this MRI compartment assessment with respect to histopathology and report the results achieved by MRI-guided beyond-TME surgery. Our hypothesis is that the number of resected compartments is not associated with prognosis if the MRI assessment is accurate.

## 2. Materials and Methods

**Figure 1:**
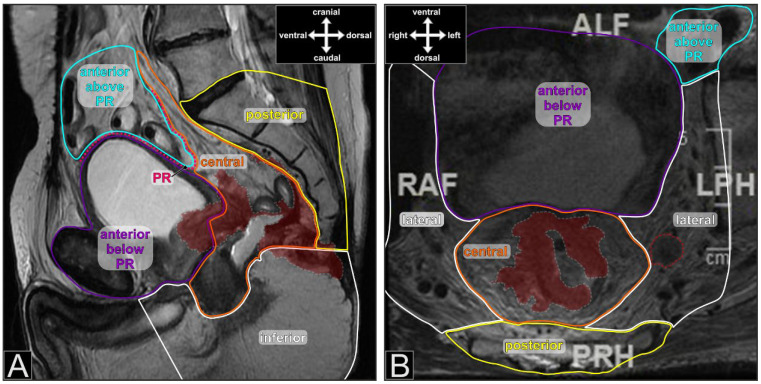
Pelvic MRI with delineation of the pelvic compartments (different colors, T2-weighted fast spin echo sequence). (**A**) sagittal image with a locally recurrent rectal cancer (anastomotic recurrence in a 68-year-old male) after anterior resection. The recurrent tumor is delineated with a grey dotted line and colored burgundy. PR—peritoneal reflection; (**B**) axial image with a cT3 primary tumor (marked as in A) and a positive lymph node in the left lateral compartment (red dotted line) in a 70-year-old male. The lymph node proved to be infiltrated by adenocarcinoma on histopathology after RCT and en bloc resection of the central and left lateral compartments.

The database of the colorectal unit of Dresden-Friedrichstadt General Hospital was queried for all consecutive patients with resection of rectal cancer infiltrating adjacent organs or exhibiting positive lateral lymph nodes (Figure 1B) that required en bloc resection of the lateral pelvic compartment (primary tumor group). Additionally, all patients operated on for a local recurrence of rectal cancer were retrieved (local recurrence group). The chosen time interval ranged from 2005 to 2019. Inclusion criteria were histologically confirmed adenocarcinoma, resection of either an adjacent organ or en bloc resection of one or both lateral compartments and attempt of complete tumor removal. Patients with histology other than adenocarcinoma or without an MRI of the pelvis were excluded. The extracted data were supplemented by an extensive chart review. We documented patient, treatment, and tumor characteristics. Additionally, initial MRI scans and reports were reviewed with respect to the extent of infiltration according to the seven compartments described by Georgiou et al. (Figure 1) [11]. If tumor infiltration was detected within the confines of one compartment, the compartment was judged infiltrated irrespective of the extent of infiltration. Investigators of the MRI scans were blinded against the pathology reports. Likewise, all histopathology reports were screened for the description of adjacent organ infiltration, and the declaration of compartment involvement followed the definitions of Georgiou et al. [11]. If a compartment was described as positive in MRI and negative in histopathology, the combination was judged as overstaging; likewise, if a compartment was negative in MRI and positive in histopathology, it was declared as understaging.

All patients were discussed in a multidisciplinary team (MDT) and offered radiochemotherapy for downsizing whenever possible. As a rule, a second pelvic MRI was performed after preoperative therapy in order to document tumor response. It was, however, not considered for the assessment of this study.

MRI examinations were performed with a 1.5-Tesla General Electrics scanner (General Electrics Company, Boston, MA, USA). According to protocol, two phased-array surface coils equipped with four receiving channels were employed for signal detection. The positioning of the coils was on the pelvis and underneath the patient. For gross orientation, a sagittal T2-weighted turbo spin-echo was used in order to detect the tumor location. In primary rectal cancer, the protocol followed the recommendations of the MERCURY study, including high-resolution T2 fast relaxation fast spin echo images perpendicular to the longitudinal axis of the rectum [13,14]. For these images, a small field of view (20 cm) and a slice thickness of 3 mm (gap 0.3 mm) were chosen. Scan acquisition parameters were: echo time (TE) 110.0 ms, repetition time (TR) 3357.0 ms, Echo Train Length (ETL) 15, and Receiver Bandwidth 31.25 kHz. Neither contrast agents nor diffusion-weighted imaging (DWI) were systematically employed in the considered time period. In local recurrence, image acquisition was tailored to the tumor location in the turbo spin-echo images. 

All patients were treated by laparotomy. The extent of the operation was guided by the pretherapeutic MRI imaging and intraoperative assessment. If performed, pelvic side wall dissection was done en bloc, usually with resection of the internal iliac vessels of the involved side [15]. Further details of anatomical landmarks and surgical strategies have only recently been described elsewhere [12,16].

Follow-up was realized in our outpatient clinic with at least annual visits and appropriate investigations as recommended by the German guidelines [17]. A detailed description has formerly been given [18].

All parameters were compared for the two groups. MRI findings were analyzed with regard to the pathology findings as the gold standard. We evaluated accuracy, specificity, sensitivity, negative predictive value, and positive predictive value for all compartments and repeated the analysis for every single compartment. As appropriate, patient, treatment, and tumor characteristics were compared with the χ2-test, Fisher exact test, and Mann–Whitney-U test. Survival was calculated as overall survival (OS) according to Kaplan–Meier and potential prognostic factors were tested with the log-rank test. These potential prognosticators were included in a multivariate Cox proportional model to elicit independent associations. The starting point for survival analysis was the date of multivisceral resection. Death of any cause was counted as an event. Patients who were lost to follow-up or had less than 60 months of observation time at the closing date of the study (31 March 2023) were censored. A *p*-value of <0.05 was considered significant. Statistical analysis was performed with SPSS^®^ version 29 (IBM Corp., Armonk, NY, USA).

## 3. Results

**Figure 2:**
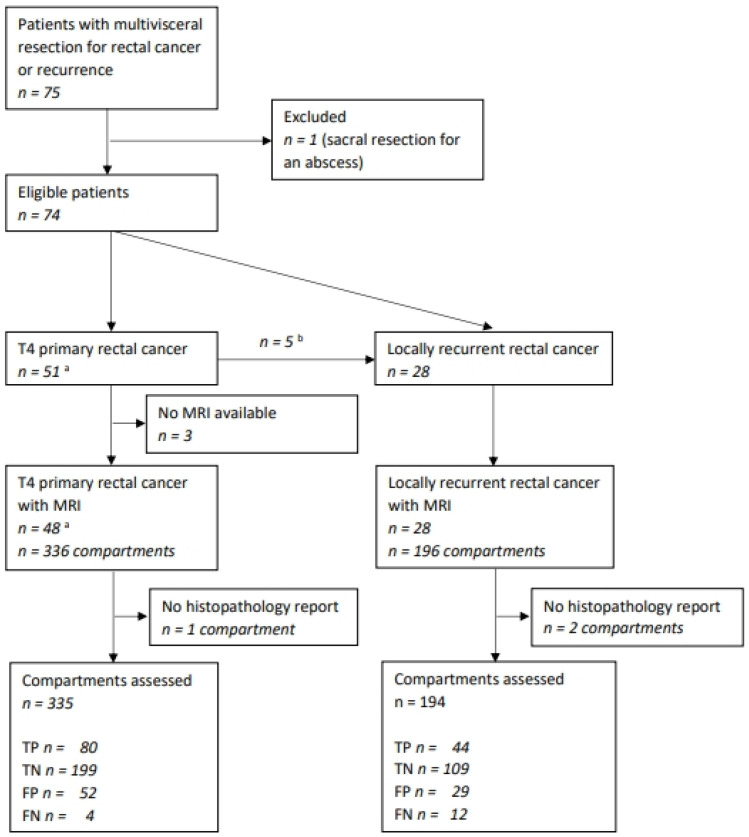
Flow chart of the study population/MRI assessment. TP—true positive, TN—true negative, FP—false positive, FN—false negative. ^a^—including two cT3 tumors with positive lateral lymph nodes; ^b^—five patients were operated on for a local recurrence after multivisceral resection for a T4 primary tumor and were investigated in both groups.

We identified 75 consecutive patients with a multivisceral resection in the predefined time period. Four patients were excluded because of a missing MRI (*n* = 3) and a sacral resection for an abscess (*n* = 1), leaving 71 to be considered. Five patients recurred after initial multivisceral resection and underwent further exenterative surgery for their recurrence. They were analyzed in both groups; thus, 76 cases (25 (32.9%) females) were included in the analysis. Forty-eight patients (63.2%) were operated for their primary tumor (including two cT3 tumors with positive lateral lymph nodes) and 28 (36.8%)) for a LRRC (Figure 2). Median age was 66.5 (interquartile range (IQR) 58–73) years, with patients in the LRRC group slightly older than in the primary tumor group (68 vs. 65 years). Median follow-up for surviving patients was 72.6 months, with only one patient lost. Further patients and tumor characteristics are given in Table 1.

A median of 3 compartments were involved in both groups on MRI. However, significantly more compartments were resected in patients with recurrent disease (median 4 (IQR 3–5) vs. 3 (IQR 3–4), *p* = 0.017). Cystectomy was performed in half of the patients with LRRC compared to one-third in those with T4 primary tumor. Vascular resections and sacral resections were performed significantly more often in LRRC, whereas significantly more hysterectomies were performed in women with T4 tumors. A pelvic floor reconstruction with a VRAM flap was significantly more often necessary in exenterative surgery for LRRC. In histopathologic work-up, a local R0-resection was achieved in 45 (93.8%) patients with primary tumors and 16 (57.1%) with recurrent tumors (*p* < 0.001). However, the median of involved compartments on histopathology was equal in both groups (2 (IQR 1–2) in primary tumor, 2 (IQR 1–3) in LRRC, *p* = 0.480) (Table 2).

Overall, 529 compartments were assessed, with three missing statements in the pathology report (Figure 2). Overall, accuracy was 81.7%, with a sensitivity of 88.6%, a specificity of 79.2%, a positive predictive value of 60.5% and a negative predictive value of 95.1%. Accuracy was somewhat higher for patients with T4 tumors compared to those with LRRC (83.3% vs. 78.9%). Likewise, sensitivity was better for patients with T4 tumors than for patients with LRRC (95.2% vs. 78.6%). Accuracy was highest for the anterior above peritoneal reflection (AAPR) compartment (90.8) and lowest for the lateral compartment (70.7). Overstaging summed up to 15.3%, whereas understaging was as low as 3.0%. Detailed figures for diagnostic performance are given in Table 3.

**Figure 3:**
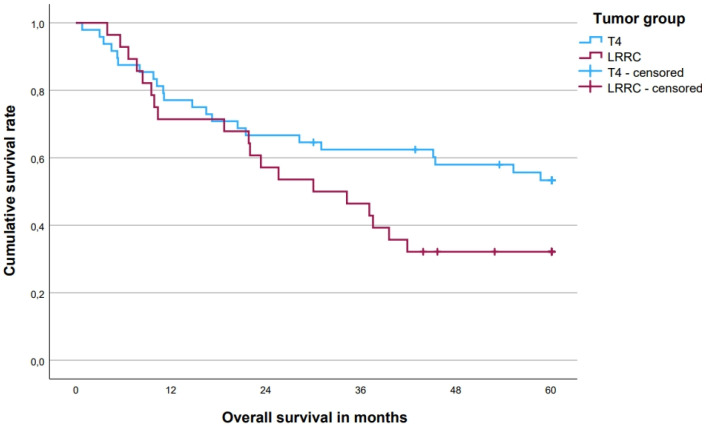
Five-year overall survival for primary tumors vs. locally recurrent rectal cancer. Five-year overall survival rates: T4 primary tumors (*n* = 48) 53.3%, locally recurrent rectal cancer (*n* = 28) 32.1% (*p* = 0.085). LRRC—locally recurrent rectal cancer.

**Figure 4:**
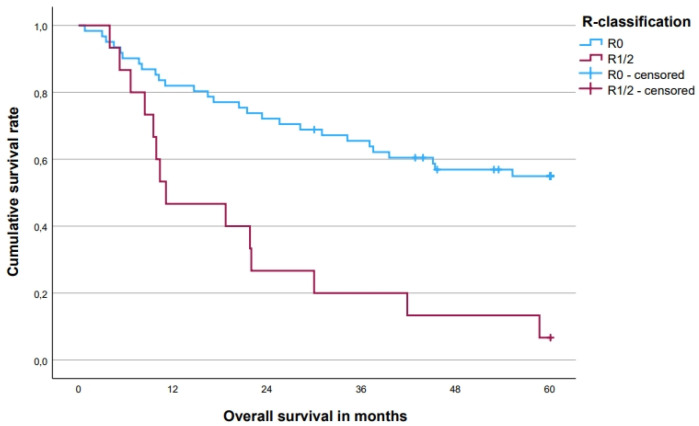
Five-year overall survival for locally R0- vs. R1/2-resected tumors. Five-year overall survival rates: R0 (*n* = 61) 54.9%, R1/2 (*n* = 15) 6.7% (*p* < 0.001).

**Figure 5:**
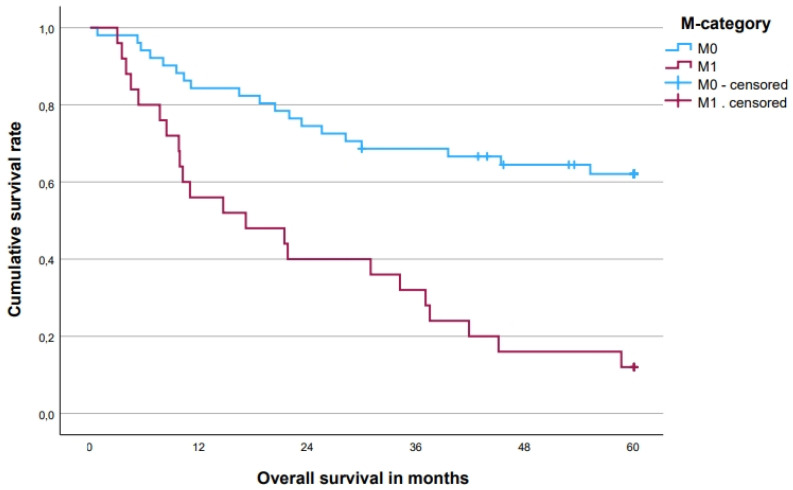
Five-year overall survival for M0 vs. M1 tumors. Five-year overall survival rates: M0 (*n* = 51) 62.1%, M1 (*n* = 25) 12.0% (*p* < 0.001).

**Figure 6:**
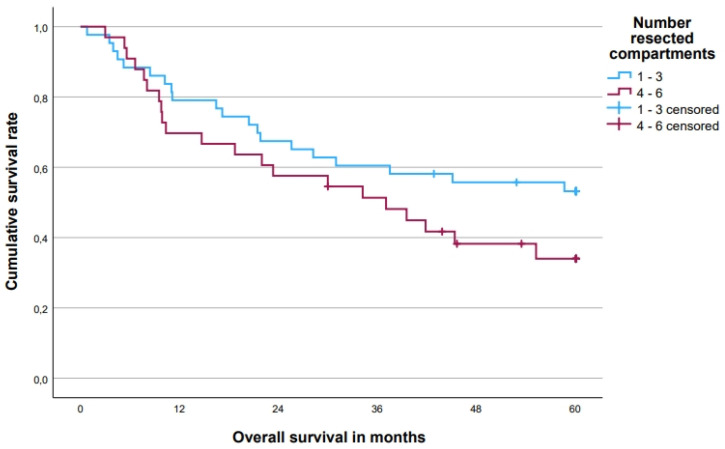
Five-year overall survival for a number of resected compartments. Five-year overall survival rates: one to three compartments (*n* = 43) 53.2%, four to six compartments (*n* = 33) 34.0% (*p* = 0.144).

The 5-year OS rate was 45.2 [33.8; 56.6 (95% CI)]% for all patients, with a difference between patients with T4-tumors (53.3%) and LRRC (32.1%, *p* = 0.085, Figure 3). In patients with a complete local pathohistological (R0) resection, the 5-year OS rate was 54.9% compared to 6.7% in patients with an R1/2 resection (*p* < 0.001, Figure 4). There was also a difference of 53.2% vs. 34.0% for patients with 1–3 vs. 4–6 resected compartments (*p* = 0.144, Figure 5). Patients without distant metastases had a clear survival advantage (Figure 6), whereas pretherapeutic CEA level was only non-significantly associated with prognosis (Table 4). In multivariate analysis, R0-resections and M0 category emerged as independent prognosticators, whereas the number of resected compartments showed no independent association with prognosis (Table 5). 

## 4. Discussion

Our study demonstrates that MRI is able to predict the status of the pelvic compartments correctly in 81.7%. The proportion of understaging, including the risk of leaving an involved compartment behind, was only 3.0%. This translates into a favorable local R0 resection rate for patients with T4 primary rectal cancer and a good R0 resection rate for patients with local recurrence. The number of resected compartments was not independently associated with prognosis in multivariate testing. This is an indicator that the attempt at resection of a tumor, which has extended beyond surgical TME planes, is warranted as long as an R0 resection seems possible. The high precision of MRI to identify involved compartments (sensitivity of 88.6%) makes this diagnostic tool the first choice in planning extended procedures.

### 4.1. Diagnostic Accuracy

Overall, the possibility of detecting an LRRC by MRI correctly was given with a sensitivity of 77–100% and a specificity of 29–86% in a recent review [19]. Data on diagnostic performance with regard to different pelvic compartments are scarce. Georgiou et al. achieved an overall accuracy of 93.1% [11]. These excellent results are attributable to their strive to keep the interval between MRI acquisition and surgery as short as possible, including post-neoadjuvant therapy imaging for assessment. Our study examines the initial MRI, which in many cases was then followed by neoadjuvant radio(chemo)therapy. Neoadjuvant therapy has the potential to downsize the tumor with the possibility of tumor withdrawal from involved tissues. However, it may be difficult to differentiate remaining fibrosis from a tumor on MRI; therefore, we planned surgery according to the pretherapeutic images [3]. If, on histopathology, no tumor was detectable, the compartment in question was counted as a false positive. This resulted in a rather high sensitivity (88.6%) but a somewhat lower specificity (79.2%). For comparison, Georgiou et al. achieved 96.0% and 90.7%, respectively [11]. The high diagnostic reliability of MRI for the absence of tumor invasion into adjacent structures was also confirmed by a Dutch group with negative predictive values between 93% and 100% [20]. The problem of fibrosis and scarring was especially evident in LRRC. Accordingly, Brown et al. examining exclusively LRRC achieved an accuracy of 82.8% with a sensitivity of 77.4% and a specificity of 85.0% [21]. Another pitfall of compartment assessment is the common recognition that posterior compartment involvement is described only on histopathology if bony infiltration can be demonstrated [22]. While tumors can often be found to have breached the posterior mesorectal fascia, an infiltration into or beyond the periost rarely occurs. Furthermore, the assessment of the lateral compartment was repeatedly reported to be problematic [11,20,21,23]. The multitude of anatomical structures and possible pathways of tumor spread along lymphatic and vascular structures may be the reason for difficulties in correct assessment [24].

### 4.2. R0-Rates

The strategy of beyond-TME surgery is to resect the adjacent compartment or at least parts of it if the tumor extends the boundaries of the mesorectal fascia and infiltrates into the compartment in question. The rationale behind it is twofold: first, to obtain a clear margin and not to risk inadvertent exposure of the tumor surface to the operation field; second, to address the possible potential pathways of further tumor spread of the adjacent compartment. The latter has hitherto not yet been fully elucidated. Whereas involvement of the lateral compartment often results from a lateral route of lymphatic spread prone to continue to more central lymphatic stations, e.g., the common iliacal or paraaortal nodes, the spread along the lymphatic or vascular routes of an involved urogenital organ or the bony pelvis remains unclear. Furthermore, the anatomical boundaries of the pelvic compartments do, in part, overlap and are not delineated as clearly as the mesorectal compartment [12]. It is, therefore, of paramount importance to surgically interpret the radiological MRI findings within multidisciplinary sessions in order to define an individual MRI-guided surgical strategy on a patient basis [10,25]. However, for comparisons of results and the determination of the case mix, a description of involved well-defined compartments remains indispensable. 

Tumor biology of primary T4 tumors and locally recurrent tumors is obviously different [26]. Primary T4 tumors represent a continuous tumor mass with compact cell formations. Clearance rates are excellent, and the prognosis is very good if no distant metastases are present. The R0 resection rates are reported to range between 72% and 91% [27,28,29]. Our rate of 93.8% compares favorably with these figures and translates into a 5-year OS rate of 53.3%. On the contrary, LRRC is disadvantaged by the fact that in the majority of cases, tumor cells have already escaped the confines of the mesorectal compartment and have inadvertently been left behind after TME surgery. Moreover, primary surgery disrupts tissue planes, restricts local blood supply, and results in scarring, all of which pave the way for diffuse and maybe discontinuous tumor spread in local recurrence. Correspondingly, an R0 resection is much more difficult to achieve with R0 rates given in the literature between 55% and 76% [26,27,30,31,32]. Our results (57.1%) are within the range of these figures, although we surgically removed significantly more compartments than in T4 tumors. Again, the lateral and the posterior compartments are repeatedly reported to set limitations to radical resection [33,34,35,36].

### 4.3. Prognosis

Given meticulous staging, multimodal treatment, and dedicated surgery, the survival rates of cT4 rectal cancer approach that of cT3 tumors. Determinants of prognosis after R0 resection are metastatic disease, pathological lymph node status, and status of the circumferential resection margin [37]. A multicenter observational study from the PelvEx Group reported a 3-year OS of R0-resected patients of 56.4% (*n* = 1030) [29]. In a large single-center study from Wales (*n* = 174), the 5-year OS was 56.4% [37]. If we restricted our analysis to overall R0 patients, the respective 3- and 5-year OS data were 64.4% and 57.1% (data not shown). These figures, however, have to be interpreted with caution because T4 tumors include tumors from stages II to IV, and survival data depend on the proportion of the different stages.

The prognosis for LRRC from the time point of recurrence detection is much worse. The aforementioned PelvEx Group analyzed 656 patients after R0 resection for local recurrence and estimated a 3-year OS of 48.1% [30]. In a meta-analysis, Banghu et al. reported 5y OS rates to range from 28 to 92% [38]. The 3- and 5-year rates of R0-resected patients with LRRC from our study population were 68.8% and 50.0% (data not shown). Again, the prognosis depends on tumor load and patient selection and has to be interpreted with caution.

The strengths of our study are the high rate of pretherapeutic MRI and an almost complete follow-up. There are some limitations which should be discussed. First, although based on a prospective database, the study is retrospective in nature, with all limitations inherent in this kind of study. However, direct comparison of MRI and histopathological data by a compartment-for-compartment base permits insights into the robustness of MRI staging in daily clinical practice. Second, more sophisticated imaging techniques like DWI were not systematically used. The MRI protocol followed the suggestions of the MERCURY group, and involved radiologists were trained as participants of the Low Rectal Cancer study [39]. Third, only resected compartments could be investigated on histopathology. There was no systematic attempt to investigate the remaining compartments after surgery. Thus, compartments judged by the surgeon to be free of tumor and left behind were counted as not involved. Fourth, the proportion of false positive compartments was rather high, in part owing to the use of pretherapeutic images for assessment. Albeit results, especially for primary T4 tumors, are favorable, a change of strategy with careful assessment of post-neoadjuvant treatment MRI and preservation of non-involved compartments deserves further evaluation.

## 5. Conclusions 

MRI is able to predict pelvic compartment involvement by T4 or LRRC tumors with high accuracy and an especially low percentage of understaging. This translates into excellent results of surgery for T4 tumors and good results for the more challenging LRRC. The number of resected compartments is not independently associated with outcomes as long as an R0 resection can be achieved.

## Figures and Tables

**Table 1:** Patient and tumor characteristics.

Parameter	Primary Tumor Group (*n* = 48)	Local Recurrence Group (*n* = 28)	Total (*n* = 76)	*p*
Age (median (IQR) in years)	65 (58–73)	68 (58–74)	66.5 (58–73)	0.477 ^a^
Sex				0.618 ^b^
Male	31 (64.6)	20 (71.4)	51 (67.1)	
Female	17 (35.4)	8 (28.6)	25 (32.9)	
Follow-up (median (IQR) in months)	72.6 (63.2–111.2)	74.3 (47.5–112.4)	72.6 (61.6–111.2)	0.674 ^a^
Pretherapeutic CEA				
Normal	22 (45.8)	12 (42.9)	34 (44.7)	0.816 ^b^
Elevated	25 (54.2)	16 (57.1)	42 (55.3)	
Values in ng/L (median (IQR) in months)	7.0 (3.0–41.8)	8.6 (2.1–24.8)	7.9 (3.0–32.0)	
Tumor extent				1.000 ^b^
(r)cT0–3 ^c^	2 (4.2)	1 (3.6)	3 (3.9)	
(r)cT4	46 (95.8)	27 (100)	73 (97.3)	
Lateral lymph nodes				0.142 ^b^
No	40 (83.3)	27 (96.4)	67 (88.2)	
Yes	8 (16.7)	1 (3.6)	9 (11.8)	
Distant metastases				1.000 ^b^
No	32 (66.7)	19 (67.9)	51 (67.1)	
Yes	16 (33.3)	9 (32.1)	25 (32.9)	
Preoperative irradiation of the small pelvis				0.001 ^b^
No	7 (14.6)	14 (50.0)	21 (27.6)	
Yes	41 (85.4)	14 (50.0)	55 (72.4)	

Values in parentheses are percentages if not otherwise specified. IQR—interquartile range; CEA—carcinoembryonic antigen. ^a^—Mann–Whitney-U test, ^b^—Fisher exact test, ^c^—patients with lateral lymph nodes.

**Table 2:** Operative, histopathologic, and MRI characteristics.

Parameter	Primary Tumor Group (*n* = 48)	Local Recurrence Group (*n* = 28)	Total (*n* = 76)	*p*
Resected organs				
Cystectomy	15 (31.3)	14 (50.0)	29 (38.2)	0.143 ^a^
partial resection of the bladder	2 (4.2)	0	2 (2.6)	0.528 ^a^
Hysterectomy ^b^	14 (82.4)	1 (12.5)	15 (60.0)	0.002 ^a^
Vaginal resection ^b^	9 (52.9)	6 (75.0)	15 (60.0)	0.402 ^a^
Vascular resection	8 (16.7)	12 (42.9)	20 (26.3)	0.016 ^a^
Sacral resection	2 (4.2)	7 (25.0)	9 (11.8)	0.010 ^a^
en bloc resection lateral compartment	25 (52.1)	19 (67.9)	44 (57.9)	0.231 ^a^
Flap reconstruction				<0.001 ^c^
none	38 (79.2)	11 (39.3)	49 (64.5)	
V-Y	5 (10.4)	3 (10.7)	8 (10.5)	
VRAM	5 (10.4)	14 (50.0)	19 (25.0)	
(r)pT-category ^d^				
0	1 (2.1)	2 (7.1)	3 (3.9)	
1	1 (2.1)	0	1 (1.3)	
2	3 (6.3)	1 (3.6)	4 (5.3)	
3	21 (43.8)	2 (7.1)	23 (30.3)	
4	22 (45.8)	23 (82.1)	45 (59.2)	0.003 ^a,e^
(r)pN-category ^d^				0.044 ^c^
0	27 (56.3)	22 (78.6)	49 (64.5)	
1	13 (27.1)	6 (21.4)	19 (25.0)	
2	8 (16.7)	0	8 (10.5)	
R-classification (local)				<0.001 ^a^
0	45 (93.8)	16 (57.1)	61 (80.3)	
1/2	3 (6.3)	12 ^f^ (42.9)	15 (19.7)	
Involved compartments on MRI, median (IQR)	3 (2–3)	3 (2–3)	3 (2–3)	0.717 ^g^
Resected compartments, median (IQR)	3 (3–4)	4 (3–5)	3 (3–4)	0.017 ^g^
Involved compartments on histopathology, median (IQR)	2 (1–2)	2 (1–3)	2 (1–2)	0.480 ^g^

Values in parentheses are percentages if not otherwise specified. V-Y—VY advancement flap; VRAM—vertical rectus abdominis myocutaneous flap; IQR—interquartile range. ^a^—Fisher-exact test; ^b^—female patients only; ^c^—χ^2^-test; ^d^—including yp and p categories; ^e^—(r)pT4 vs. all other categories, ^f^—two patients R2; ^g^—Mann-Withney-U test.

**Table 3:** Diagnostic performance MRI vs. histopathology.

Parameter	All-Comp.	T4-Comp.	LR-Comp.	PR	AAPR	ABPR	Central	Lateral	Posterior	Inferior
Accuracy—TP+TN/all	81.7	83.3	78.9	78.7	90.8	76.3	89.5	70.7	86.8	78.7
Sensitivity—TP/TP+FN	88.6	95.2	78.6	-	71.4	100	93.5	77.8	71.4	87.5
Specificity—TN/TN+FP	79.2	79.3	79.0	81.9	92.8	56.1	71.4	67.2	88.4	77.6
PPV—TP/TP+FP	60.5	60.0	60.3	-	50.0	66.0	93.5	43.8	38.5	31.8
NPV—TN/TN+FN	95.1	98.0	90.1	95.2	97.0	100	71.4	90.7	96.8	98.1
Overstaging FP/all	15.3	15.5	14.9	17.3	6.6	23.7	5.3	24.0	10.5	20.0
Unterstaging FN/all	3.0	1.2	6.2	4.0	2.6	0	5.3	5.3	2.6	1.3

All values are percentages. Comp.—all compartments; LR—local recurrence; PR—peritoneal reflection compartment; AAPR—anterior above peritoneal reflection compartment; ABPR—anterior below peritoneal reflection compartment; TP—true positive; TN—true negative; FP—false positive; FN—false negative; PPV—positive predictive value; NPV—negative predictive value.

**Table 4:** Five-year overall survival rates.

Parameter	*n*	5-Year Overall Survival in % [95% CI (%)]	Events	*p*
Total	76	45.2 [33.8; 56.6]	41	
Group				0.085
Primary tumor	48	53.3 [39.0; 67.6]	22	
Local recurrence	28	32.1 [14.8; 49.3]	19	
R-classification				<0.001
0	61	54.9 [42.2; 67.6]	27	
1/2	15	6.7 [0; 19.2]	14	
Number resected compartments				0.144
1–3	43	53.2 [38.1; 68.3]	20	
4–6	33	34.0 [16.9; 51.1]	21	
M-category				<0.001
0	51	62.1 [48.6; 75.6]	19	
1	25	12.0 [0; 24.7]	22	
Pretherapeutic CEA				0.112
Normal	34	54.9 [37.8; 72.0]	15	
Elevated	42	37.5 [22.6; 52.4]	26	

CI—confidence interval; CEA—carcinoembryonic antigen.

**Table 5:** Cox regression analysis for 5-year overall survival.

Parameter	Univariate			Multivariate		
	Hazard Ratio	95% CI	*p*	Hazard Ratio	95% CI	*p*
Group						
Primary tumor	Ref.			Ref.		
Local recurrence	1.712	0.923; 3.177	0.088	0.988	0.455; 2.147	0.975
R-classification						
0	Ref.			Ref.		
1/2	3.617	1.868; 7.004	<0.001	2.509	1.168; 5.391	0.018
No. resected compartments						
1–3	Ref.			Ref.		
4–6	1.575	0.852; 2.912	0.147	1.633	0.795; 3.355	0.182
Distant metastases						
M0	Ref.			Ref.		
M1	3.820	2.049; 7.123	<0.001	3.479	1.810; 6.688	<0.001
Pretherapeutic CEA						
Normal	Ref.			Ref.		
Elevated	1.667	0.882; 3.151	0.116	1.378	0.692; 2.745	0.362

CI—confidence interval; CEA—carcinoembryonic antigen.

## Data Availability

The data presented in this study are available upon reasonable request from the corresponding author.

## References

[1] Ruppert R., Junginger T., Kube R., Strassburg J., Lewin A., Baral J., Maurer C.A., Sauer J., Lauscher J., Winde G. (2023). Risk-adapted neoadjuvant chemoradiotherapy in rectal cancer: Final report of the OCUM study. J. Clin. Oncol..

[2] Jacob A., Albert W., Jackisch T., Jakob C., Sims A., Witzigmann H., Mees S.T., Stelzner S. (2021). Association of certification, improved quality and better oncological outcomes for rectal cancer in a specialized colorectal unit. Int. J. Colorectal Dis..

[3] PelvEx Collaborative (2022). Contemporary management of locally advanced and recurrent rectal cancer: Views from the PelvEx Collaborative. Cancers.

[4] Detering R., Saraste D., de Neree Tot Babberich M.P.M., Dekker J.W.T., Wouters M.W.J.M., van Geloven A.A.W., Bemelman W.A., Tanis P.J., Martling A., Westerterp M. (2020). International evaluation of circumferential resection margins after rectal cancer resection: Insights from the Swedish and Dutch audits. Colorectal Dis..

[5] The Beyond TME Collaborative (2013). Consensus statement on the multidisciplinary management of patients with recurrent and primary rectal cancer beyond total mesorectal excision planes. Br. J. Surg..

[6] Peacock O., Waters P.S., Kong J.C., Warrier S.K., Wakeman C., Eglinton T., Heriot A.G., Frizelle F.A., McCormick J.J. (2020). Complications after extended radical resections for locally advanced and recurrent pelvic malignancies: A 25-year experience. Ann. Surg. Oncol..

[7] Harji D.P., Griffiths B., Velikova G., Sagar P.M., Brown J. (2016). Systematic review of health-related quality of life in patients undergoing pelvic exenteration. Eur. J. Surg. Oncol..

[8] Inoue A., Sheedy S.P., Wells M.L., Mileto A., Goenka A.H., Ehman E.C., Yalon M., Murthy N.S., Mathis K.L., Behm K.T. (2023). Rectal cancer pelvic recurrence: Imaging patterns and key concepts to guide treatment planning. Abdom. Radiol..

[9] Ng K.S., Lee P.J.M. (2021). Pelvic exenteration: Pre-, intra-, and post-operative considerations. Surg. Oncol..

[10] PelvEx Collaborative (2022). Minimum standards of pelvic exenterative practice: PelvEx Collaborative guideline. Br. J. Surg..

[11] Georgiou P.A., Tekkis P.P., Constantinides V.A., Patel U., Goldin R.D., Darzi A.W., Nicholls J.R., Brown G. (2013). Diagnostic accuracy and value of magnetic resonance imaging (MRI) in planning exenterative pelvic surgery for advanced colorectal cancer. Eur. J. Cancer.

[12] Stelzner S., Heinze T., Heimke M., Gockel I., Kittner T., Brown G., Mees S.T., Wedel T. (2023). Beyond total mesorectal excision—Compartment-based anatomy of the pelvis revisited for exenterative pelvic surgery. Ann. Surg..

[13] Mercury Study Group (2006). Diagnostic accuracy of preoperative magnetic resonance imaging in predicting curative resection of rectal adenocarcinoma: Prospective observational study. BMJ.

[14] Brown G., Daniels I.R., Richardson C., Revell P., Peppercorn D., Bourne M. (2005). Techniques and trouble-shooting in high spatial resolution thin slice MRI for rectal cancer. Br. J. Radiol..

[15] Austin K.K., Solomon M.J. (2009). Pelvic exenteration with en bloc iliac vessel resection for lateral pelvic wall involvement. Dis. Colon. Rectum..

[16] Jackisch J., Jackisch T., Roessler J., Sims A., Nitzsche H., Mann P., Mees S.T., Stelzner S. (2022). Tailored concept for the plastic closure of pelvic defects resulting from extralevator abdominoperineal excision (ELAPE) or pelvic exenteration. Int. J. Colorectal Dis..

[17] Guideline Programme Oncology (German Cancer Society, German Cancer Aid, AWMF): Level3-Guideline Colorectal Carcinoma, Long Version 2.1, 2019, AWMF Registry No. 021/007OL. https://www.leitlinienprogramm-onkologie.de/leitlinen/kolorektales-karzinom/.

[18] Fischer J., Hellmich G., Jackisch T., Puffer E., Zimmer J., Bleyl D., Kittner T., Witzigmann H., Stelzner S. (2015). Outcome for stage II and III rectal and colon cancer equally good after treatment improvement over three decades. Int. J. Colorectal Dis..

[19] Colosio A., Fornès P., Soyer P., Lewin M., Loock M., Hoeffel C. (2013). Local colorectal cancer recurrence: Pelvic MRI evaluation. Abdom. Imaging.

[20] Dresen R.C., Kusters M., Daniels-Gooszen A.W., Cappendijk V.C., Nieuwenhuijzen G.A., Kessels A.G., de Bruïne A.P., Beets G.L., Rutten H.J., Beets-Tan R.G. (2010). Absence of tumor invasion into pelvic structures in locally recurrent rectal cancer: Prediction with preoperative MR imaging. Radiology.

[21] Brown W.E., Koh C.E., Badgery-Parker T., Solomon M.J. (2017). Validation of MRI and surgical decision making to predict a complete resection in pelvic exenteration for recurrent rectal cancer. Dis. Colon. Rectum.

[22] Yamada K., Ishizawa T., Niwa K., Chuman Y., Akiba S., Aikou T. (2001). Patterns of pelvic invasion are prognostic in the treatment of locally recurrent rectal cancer. Br. J. Surg..

[23] Messiou C., Chalmers A.G., Boyle K., Wilson D., Sagar P. (2008). Pre-operative MR assessment of recurrent rectal cancer. Br. J. Radiol..

[24] Heinze T., Fletcher J., Miskovic D., Stelzner S., Bayer A., Wedel T. (2023). The middle rectal artery: Revisited anatomy and surgical implications of a neglected blood vessel. Dis. Colon. Rectum.

[25] Akash M., Mehta D.B., Jenkins J.T. (2021). Preoperative assessment of tumor anatomy and surgical resectability. Surg. Manag. Adv. Pelvic Cancer.

[26] van Kessel C.S., Solomon M.J. (2022). Understanding the philosophy, anatomy, and surgery of the extra-TME plane of locally advanced and locally recurrent rectal cancer; single institution experience with international benchmarking. Cancers.

[27] Lau Y.C., Jongerius K., Wakeman C., Heriot A.G., Solomon M.J., Sagar P.M., Tekkis P.P., Frizelle F.A. (2019). Influence of the level of sacrectomy on survival in patients with locally advanced and recurrent rectal cancer. Br. J. Surg..

[28] Bhangu A., Ali S.M., Brown G., Nicholls R.J., Tekkis P. (2014). Indications and outcome of pelvic exenteration for locally advanced primary and recurrent rectal cancer. Ann. Surg..

[29] PelvEx Collaborative (2019). Surgical and survival outcomes following pelvic exenteration for locally advanced primary rectal cancer: Results from an international collaboration. Ann. Surg..

[30] PelvEx Collaborative (2018). Factors affecting outcomes following pelvic exenteration for locally recurrent rectal cancer. Br. J. Surg..

[31] Bird T.G., Ngan S.Y., Chu J., Kroon R., Lynch A.C., Heriot A.G. (2018). Outcomes and prognostic factors of multimodality treatment for locally recurrent rectal cancer with curative intent. Int. J. Colorectal Dis..

[32] Nordkamp S., Voogt E.L.K., van Zoggel D.M.G.I., Martling A., Holm T., Jansson Palmer G., Suzuki C., Nederend J., Kusters M., Burger J.W.A. (2022). Locally recurrent rectal cancer: Oncological outcomes with different treatment strategies in two tertiary referral units. Br. J. Surg..

[33] Belli F., Sorrentino L., Gallino G., Gronchi A., Scaramuzza D., Valvo F., Cattaneo L., Cosimelli M. (2020). A proposal of an updated classification for pelvic relapses of rectal cancer to guide surgical decision-making. J. Surg. Oncol..

[34] Iversen H., Martling A., Johansson H., Nilsson P.J., Holm T. (2018). Pelvic local recurrence from colorectal cancer: Surgical challenge with changing preconditions. Colorectal Dis..

[35] Rahbari N.N., Ulrich A.B., Bruckner T., Münter M., Nickles A., Contin P., Löffler T., Reissfelder C., Koch M., Büchler M.W. (2011). Surgery for locally recurrent rectal cancer in the era of total mesorectal excision: Is there still a chance for cure?. Ann. Surg..

[36] van Ramshorst G.H., O’Shannassy S., Brown W.E., Kench J.G., Solomon M.J. (2018). A qualitative study of the development of a multidisciplinary case conference review methodology to reduce involved margins in pelvic exenteration surgery for recurrent rectal cancer. Colorectal Dis..

[37] Radwan R.W., Jones H.G., Rawat N., Davies M., Evans M.D., Harris D.A., Beynon J., Swansea Pelvic Oncology Group (2015). Determinants of survival following pelvic exenteration for primary rectal cancer. Br. J. Surg..

[38] Bhangu A., Ali S.M., Darzi A., Brown G., Tekkis P. (2012). Meta-analysis of survival based on resection margin status following surgery for recurrent rectal cancer. Colorectal Dis..

[39] Battersby N.J., How P., Moran B., Stelzner S., West N.P., Branagan G., Strassburg J., Quirke P., Tekkis P., Pedersen B.G. (2016). Prospective Validation of a Low Rectal Cancer Magnetic Resonance Imaging Staging System and Development of a Local Recurrence Risk Stratification Model: The MERCURY II Study. Ann. Surg..

